# Automatic steel labeling on certain microstructural constituents with image processing and machine learning tools

**DOI:** 10.1080/14686996.2019.1610668

**Published:** 2019-06-05

**Authors:** Dmitry S. Bulgarevich, Susumu Tsukamoto, Tadashi Kasuya, Masahiko Demura, Makoto Watanabe

**Affiliations:** a Research and Services Division of Materials Data and Integrated System, National Institute for Materials Science, Tsukuba, Japan; b School of Engineering, The University of Tokyo, Tokyo, Japan

**Keywords:** Metallurgy, machine learning, microstructures, optical microscopy, pattern recognition, 10 Engineering and structural materials, 106 Metallic materials, 404 Materials informatics / Genomics, 505 Optical / Molecular spectroscopy

## Abstract

It is demonstrated that optical microscopy images of steel materials could be effectively categorized into classes on preset ferrite/pearlite-, ferrite/pearlite/bainite-, and bainite/martensite-type microstructures with image pre-processing and statistical analysis including the machine learning techniques. Though several popular classifiers were able to get the reasonable class-labeling accuracy, the random forest was virtually the best choice in terms of overall performance and usability. The present categorizing classifier could assist in choosing the appropriate pattern recognition method from our library for various steel microstructures, which we have recently reported. That is, the combination of the categorizing and pattern-recognizing methods provides a total solution for automatic quantification of a wide range of steel microstructures.

## GRAPHICAL ABSTRACT

**Figure:**
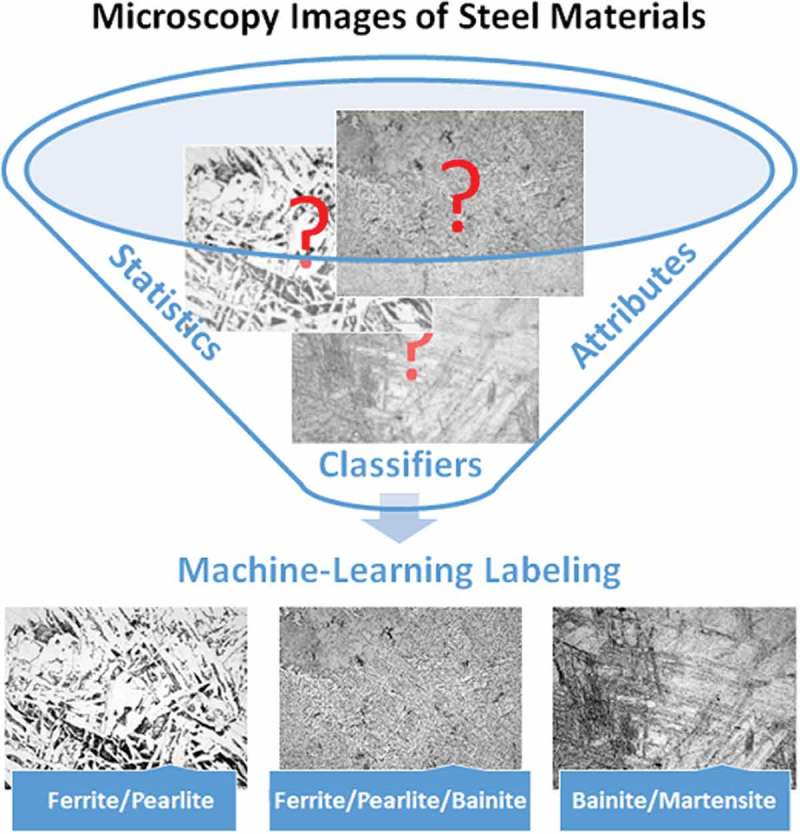

## 1. Introduction

Microstructure phase analysis is one of the primary interests in metallurgy because formed microstructures significantly determine the material properties [1–3]. Most of the data for such studies are coming from optical- or electron microscopy imaging techniques. By using modern industrial optical microscopes with advanced automated imaging equipment, scanning stages, and even sample slicing for three-dimensional imaging, the amount of image data become overwhelming for manual examinations, and, in some cases, the relevant information could be hidden even for an expert. In this respect, there are big hopes that machine learning (ML) techniques could assist in automatization of routine tasks on big image datasets and that information gains could even unveil the new material paradigms. For metallurgy, the progress in such a direction has the paramount importance. So far, however, the reported image analyses with ML on metallic materials were extremely limited and, in most cases, were not compared/matched to the standard analytical methods in the metallurgical industry [4]. This situation is in strong contrast with an explosive number of scientific publications on ML applications to material science problems by using the physicochemical and structural property datasets/databases [5–9] as well as other knowledge sources [10,11].

Consequently, we have recently reported the successful application of random forest (RF) ML classifiers to segment the optical microscopy images of typical steel materials on ferrite (F), pearlite (P), bainite (B), and martensite (M) microstructures, with possibilities of further segmentation/analysis of their corresponding sub-phases [4]. The key point of this technique was the possibility to get the excellent quality of quantitative results, which was comparable or even better than manual estimations by experts, not to mention the benefits of microstructure phase area visualizations, analysis speed boosts, and automatizations with computers.

As with any single techniques, there were also some limitations of our previously established RF classifiers. One of them was that number and type of microstructure phases/classes (j=A,B,C,...,n)present in training image dataset must correspond to the same set of j in dataset being analyzed in order to get the correct results with the specific RF classifier. This poses less of a problem for an expert who recognizes well the type of j in images for segmentation, but for any use in a large scale of industrial analytical laboratory or for the use by a non-expert in steel/metal microstructures, this limitation should be properly addressed. The prime aim of the current work is to develop additional image processing/analysis protocols for assisting in choosing the appropriate RF classifier to the image of interest.

**Figure 1.**
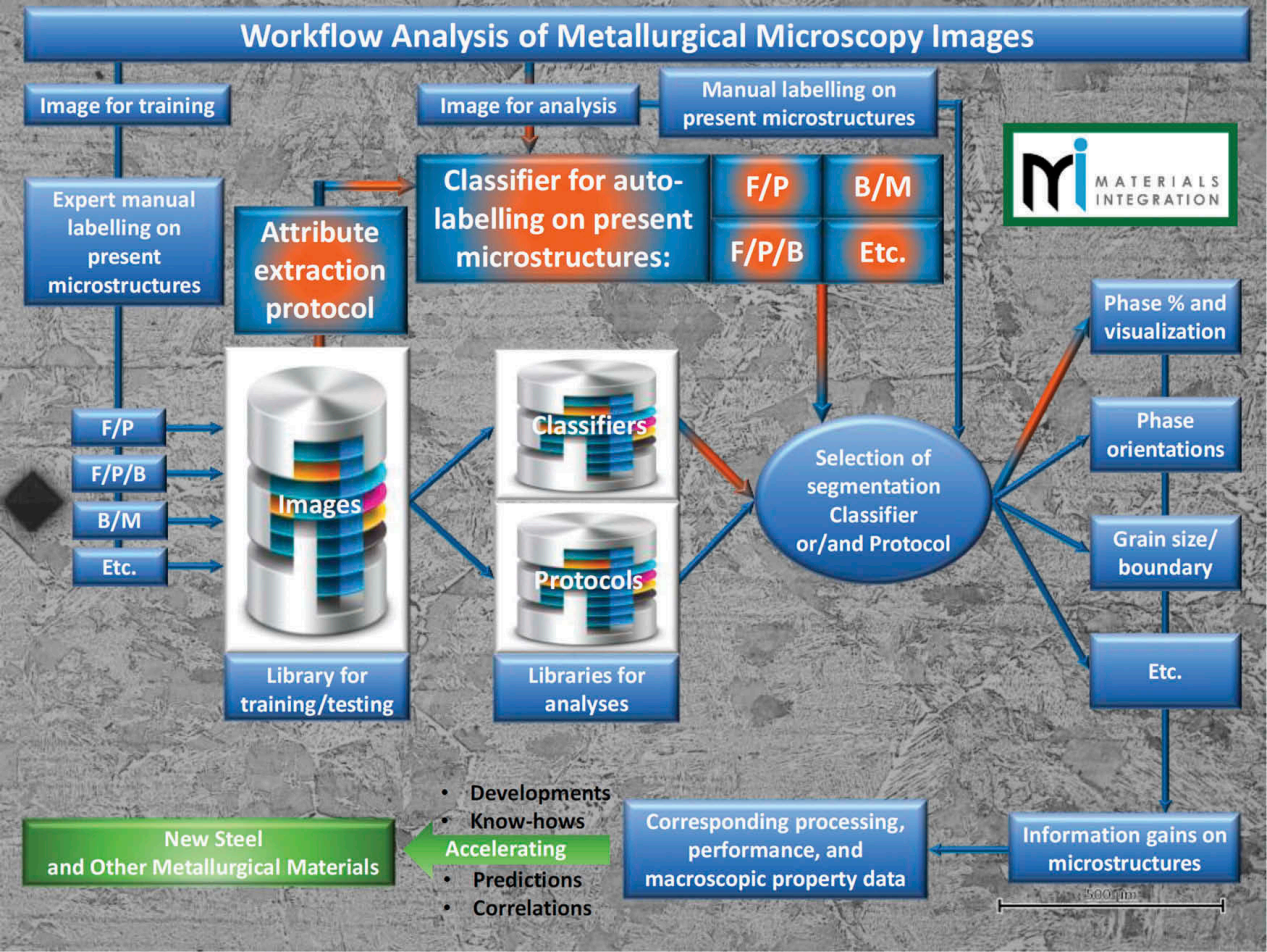
General outline of the workflow for the development of new metallurgical materials with image analysis protocols by using machine learning and image processing tools. The orange highlighted arrows and spotlighted text boxes indicate the main theme of the current work.

Figure 1 shows the envisioned general workflow of image analysis for metallic materials with ML and image processing tools. The current analysis module and its contribution to the workflow are shown by highlighted/spotlighted orange color. In the case of the human expert in metallurgy field, it is often enough to have just a brief look at the microscopy image of steel material for judging what types of microstructures, e.g. F/P-, F/P/B-, or B/M-type ones, the image shows. To do it on a computer in an automated way could bring the great benefits, for example, in analysis of a welded part of steels that should contain all sorts of the microstructure types at different areas. The high-resolution image data of the whole welded part could contain thousands of images, which hold a lot of useful information, but which are difficult/impractical to sort and analyze manually. This is our first attempt to address this problem.

## 2. Experimental details

**Figure 2.**
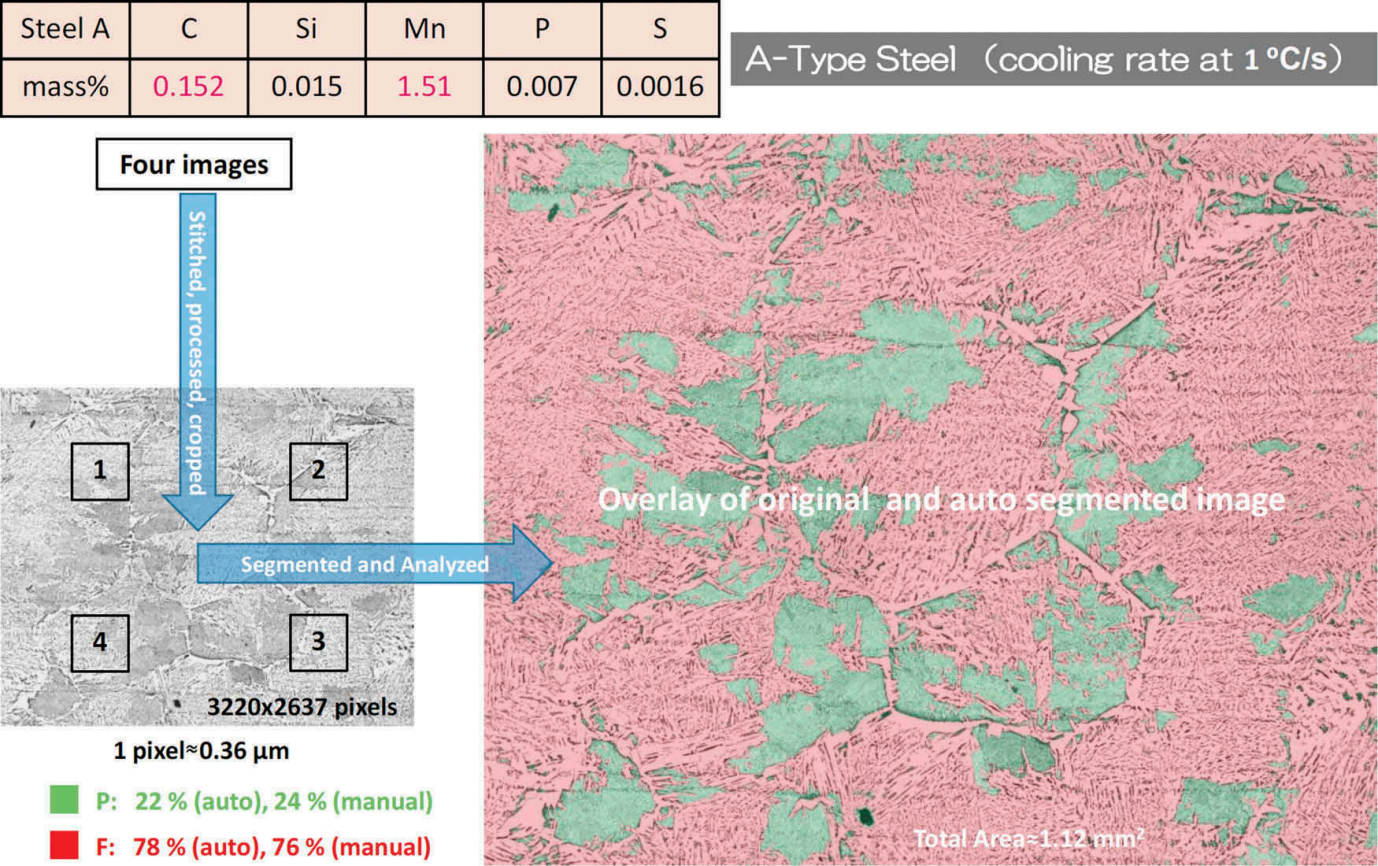
Example of image segmentation protocol on F and P microstructure phases by using the image pre-processing and random forest classifier tools. The color coding on F and P microstructure areas of A-type steel is given on insert together with results of quantitative manual and automated analyses.

All samples of A-type steel (see composition in Figure 2) obtained by cooling of this alloy from 1400°C at 0.3, 1, 3, and 10°C/s rates were prepared by a conventional polishing and subsequent etching with 0.5% picric and 0.5% nitric acids in ethanol [12]. The microstructures in our samples correspond to the typical ones appearing in a welded part of steels. They were imaged with same spatial resolution on BX53M optical microscope (Olympus, Japan) equipped with MPLN50x objective (Olympus, Japan) and DP22 CCD camera (Olympus, Japan), which satisfied the pixel size requirement for maximum optical resolution (see grayscale image in Figure 2). The samples at 0.3, 1, 3, and 10°C/s rates were identified as (F_gb_+F_all_+F_sp_)/P, (F_gb_+F_all_+F_sp_)/P, (F_all_+F_sp_)/P/B, and B/M-type microstructures, respectively, with different contributions of ferrite sub-phases: allotriomorphic (F_all_), grain boundary polygonal (F_gb_), and side plate (F_sp_) ferrites.

The image pre-processing, analysis, and segmentation were conducted by using the open-source FIJI software package with Trainable Weka Segmentation plugin on two-CPU 6128 Optiron Workstation with 128 GB RAM [13–17]. The ML on training/test datasets with statistical image data for corresponding image labeling on present microstructures was performed by using the open-source WEKA software package, which is a collection of ML algorithms for data mining tasks [18].

## 3. Results and discussion

Before discussing image labeling, Figure 2 shows the example of the application of the RF ML algorithm to train it on four microscopy optical images of A-type steel and segment them on F and P microstructure phases, which are formed by cooling from 1400°C at 1°C/s rate. The successful application of automatic pattern recognition with such well-trained RF classifier was confirmed by an expert with manual visual inspection and line-intercept analysis (see percentage numbers in Figure 2). In principle, such RF classifier could be applied to any number of other images containing F and P microstructures if such images have similar quality and same spatial resolution.

**Figure 3.**
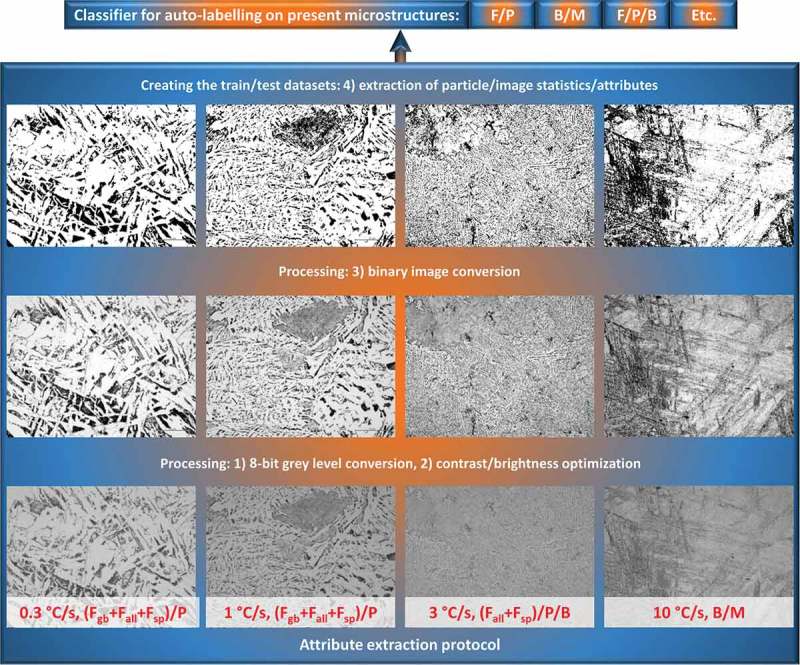
Extraction of attributes for training/test datasets in order to build the classifier for image auto-labeling on different steel types characterized by microstructure phase combinations (see text for more details).

However, as discussed in Section 1, the required prior knowledge on steel microstructures (i.e. the type of microstructures in the image of interest) could be problematic for choosing the RF classifier correctly. As one possibility to solve this problem, we extracted the statistical attributes from image datasets for various cooling rates and tried to classify/label each image into F/P-, F/P/B-, and B/M-type microstructures with ML tools. Figure 3 shows the protocol of image processing and subsequent attribute extraction: (1) automatic conversion of color image stacks to 8-bit gray level ones for each A-steel cooling rate; (2) consistent automatic optimization of brightness and contrast of resulted image stacks based on analysis of the image histograms; (3) automatic conversion of resulted image stacks to binary ones (black and white) by analyzing image histograms and subsequent thresholding; and (4) automatically count and measure the black objects/particles in stacks of resulted binary images.

Table 1 lists the names and descriptions of absolute or mean values of estimated attributes for each image (one data point) in resulted training and test datasets. In total, there were 117 and 73 of training and test data points/images in 21-dimensional attribute space to work with, respectively. They were used to train and test the different ML classifiers in order to classify into the four types of microstructures. Note that some attributes in Table 1 were completely irrelevant to our classification problem, redundant, and correlated to others. They were left on purpose and used to check the attribute selection/reduction tools and robustness of different classifiers to such datasets. This behavior is important to know and consider if adding new attributes in the future will be necessary or they will become available.

**Table 1.** Attributes in training and test datasets corresponding to single image characteristics.

No.	Attribute	Definition	Comment
1	Count	Black objects	Absolute value, correlated
2	Total area	Black objects	Absolute value, correlated
3	Size	Black objects	Mean value
4	%Area	Black objects	Absolute value, correlated
5	Mean	Average value of all pixels in black objects	Constant value, irrelevant
6	Mode	Most frequently occurring value of pixels in black objects	Constant value, irrelevant
7	Perimeter	Black objects	Mean value
8	Major	Axis of fitted ellipse into black particle	Mean value
9	Minor	Axis of fitted ellipse into black particle	Mean value
10	Angle	Angle between the fitted ellipse axis of the black particle and a line parallel to the X-axis of the image	Mean value, irrelevant
11	Circularity	Black objects, $4\pi \times \left[Size\right]/\left[Perimeter\right]$	Mean value, robust to image scale
12	Solidity	Black objects, $4\pi \times \left[Size\right]/\left[Convex\;area\right]$	Mean value, dimensionless, robust to image scale
13	Feret	Maximum caliper of black objects	Mean value
14	FeretX	Starting X coordinates of the Feret	Mean value, irrelevant
15	FeretY	Starting Y coordinates of the Feret	Mean value, irrelevant
16	FeretAngle	The angle of the Feret	Mean value, irrelevant
17	MinFeret	Minimum caliper of black objects	Mean value
18	IntDen	The sum of the values of the pixels in black objects	Mean value, correlated
19	Aspect ratio	$\left[Major\right]/\left[Minor\right]$	Mean value, dimensionless, robust to image scale
20	Density	$\left[Count\right]/\left[Total\;Area\right]$	Mean value, correlated
21	Surface density	$\left[Count\right]/\left[\mu m^{2}\right]$	Mean value, robust to image scale, correlated

Some differences between training and test datasets were also added to model the possible real-world applications: (1) different samples for test dataset were prepared time apart from training dataset, but with the same composition and cooling rates; (2) the data in test dataset for 3°C/s cooling rate were extracted from images with bad quality of sample etching for B-phase visualization; and thus, F and P phases were mainly visible in those images. Such data were used on purpose in order to check the sensitivity of attributes and classifiers to data quality variations, which could be typically encountered in actual experiments.

**Figure 4.**
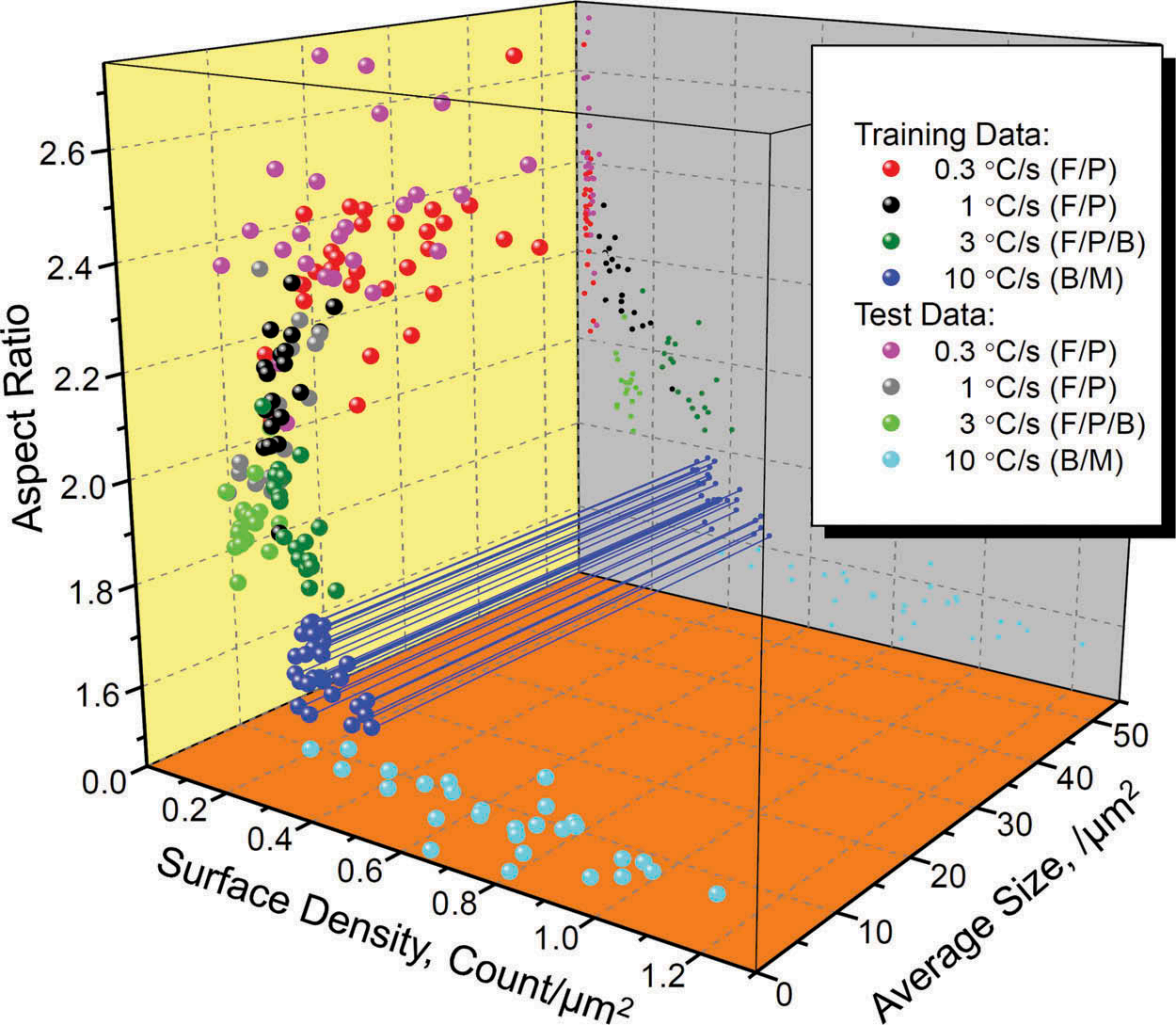
The 3D/2D slices of the multidimensional attribute space for training and test datasets of image statistics. Each attribute data point corresponds to the absolute or mean value for a single image (see Figure 3 and Table 1). The mapping example of 3D points to the 2D plane is shown for clarity with blue color lines. Additional 2D slices in the form of partial scatter matrix are available as Supplemental Material.

Figure 4 shows the examples of 3D and 2D slices of such 21-dimensional attribute space. In spite of considerable scattering, there is a visible tendency for data points in training and test datasets to group depending on types of microstructure combinations. It is also seen in the 2D slice of surface density vs. aspect ratio (plotted on gray plane) that largest deviations between training (dark green) and test data (light green) are for 3°C/s cooling rate ones, which is reasonable due to bad etching quality mentioned above. This can also be observed on 3D/2D slices for different attributes (see Supplemental Material). Then, we have applied several ML techniques to train, classify, and validate the resulted classification accuracy with them. For comparison, Table 2 lists the used methods and results of 10-fold cross-validation on the training dataset, which was utilized to build classifiers. On the next step, such classifiers were applied on test dataset for additional validations. The corresponding results are listed in Table 2.

**Table 2.** Performance comparisons between random forest, neural network, and Auto-WEKA classifiers. The *a, b, c*, and *d* columns/row labels correspond to the instances of the classification on 0.3, 1, 3, and 10°C/s or (F_gb_+F_all_+F_sp_)/P, (F_gb_+F_all_+F_sp_)/P, (F_all_+F_sp_)/P/P/B, and B/M classes, respectively. The numbers in brackets are calculated by omitting the data at 0.3°C/s. All attributes from Table 1 were used.

Classifier	Main details	Ten-fold cross-validation on training dataset	Validation on test dataset
		Confusion matrix, correct classification	Confusion Matrix, Correct classification
Random forest 0.1 s to build	Number of trees: 100 Number of randomly chosen attributes: int$\left[log_{2}\left(Attributes\right)+1\right]$	$\begin{array}{ccccc} a & b & c & d & \text{.}\text{.}\text{.}\\ 30 & 0 & 0 & 0 & a\\ 0 & 38 & 0 & 0 & b\\ 0 & 1 & 18 & 0 & c\\ 0 & 0 & 1 & 29 & d \end{array}$	$\begin{array}{ccccc} a & b & c & d & \text{.}\text{.}\text{.}\\ 23 & 1 & 0 & 0 & a\\ 0 & 16 & 0 & 0 & b\\ 0 & 10 & 5 & 2 & c\\ 0 & 0 & 0 & 29 & d \end{array}$
		~98.3%	~84.9% (98.6%)
Multilayer perceptron (neural networks) 1.0 s to build	Classifier: *Multilayer Perceptron* Momentum: 0.2 Learning rate: 0.3 One single hidden layer, number of neurons: (attributes + classes)/2	$\begin{array}{ccccc} a & b & c & d & \text{.}\text{.}\text{.}\\ 29 & 1 & 0 & 0 & a\\ 0 & 38 & 0 & 0 & b\\ 0 & 1 & 17 & 1 & c\\ 0 & 0 & 1 & 29 & d \end{array}$	$\begin{array}{ccccc} a & b & c & d & \text{.}\text{.}\text{.}\\ 23 & 1 & 0 & 0 & a\\ 5 & 11 & 0 & 0 & b\\ 0 & 17 & 0 & 0 & c\\ 0 & 0 & 0 & 29 & d \end{array}$
		~96.6%	~73.3% (91.3%)
Auto-WEKA 15 min to build	Best classifier: *Attribute Selected Classifier* Arguments: *Greedy Stepwise, Correlation-based* *Feature Subset Selection, Random Forest*	$\begin{array}{ccccc} a & b & c & d & \text{.}\text{.}\text{.}\\ 30 & 0 & 0 & 0 & a\\ 0 & 38 & 0 & 0 & b\\ 0 & 0 & 19 & 0 & c\\ 0 & 0 & 0 & 30 & d \end{array}$	$\begin{array}{ccccc} a & b & c & d & \text{.}\text{.}\text{.}\\ 22 & 2 & 0 & 0 & a\\ 0 & 16 & 0 & 0 & b\\ 0 & 17 & 0 & 0 & c\\ 0 & 0 & 0 & 29 & d \end{array}$
		100%	~68.6% (97.1%)
Auto-WEKA 12 h to build	Best classifier: *Logistic Model Trees* Arguments: *Greedy Stepwise, Correlation-based* *Feature Subset Selection*	$\begin{array}{ccccc} a & b & c & d & \text{.}\text{.}\text{.}\\ 30 & 0 & 0 & 0 & a\\ 0 & 38 & 0 & 0 & b\\ 0 & 0 & 19 & 0 & c\\ 0 & 0 & 0 & 30 & d \end{array}$	$\begin{array}{ccccc} a & b & c & d & \text{.}\text{.}\text{.}\\ 23 & 1 & 0 & 0 & a\\ 1 & 15 & 0 & 0 & b\\ 0 & 16 & 0 & 1 & c\\ 0 & 0 & 8 & 21 & d \end{array}$
		100%	~68.6% (85.5%)
Auto-WEKA 24 h to build	Best classifier: *Locally Weighted Learning* Arguments: *Greedy Stepwise, Correlation-based Feature Subset Selection, Simple Logistic*	$\begin{array}{ccccc} a & b & c & d & \text{.}\text{.}\text{.}\\ 30 & 0 & 0 & 0 & a\\ 0 & 38 & 0 & 0 & b\\ 0 & 0 & 19 & 0 & c\\ 0 & 0 & 0 & 30 & d \end{array}$	$\begin{array}{ccccc} a & b & c & d & \text{.}\text{.}\text{.}\\ 23 & 1 & 0 & 0 & a\\ 0 & 16 & 0 & 0 & b\\ 0 & 14 & 2 & 1 & c\\ 0 & 0 & 8 & 21 & d \end{array}$
		100%	~81.4% (87.0%)

In addition to RF, multilayer perceptron (MP), and other individual classifiers, we also used the state-of-the-art Auto-WEKA, which is the RF-based Bayesian optimization module for tuning hyperparameters of the classification algorithms built into the WEKA software package [19,20]. It works with 27 base ML algorithms, 10 meta- and 2 ensemble-methods, combining 3 search and 8 evaluator techniques. In total, 789 hyperparameters from all classification algorithms and feature selectors/evaluators could be used in optimization depending on accuracy, time, and PC resources. Table 2 lists several Auto-WEKA runs with names of classifiers and main arguments, which is selected for the given computation time. Note that Auto-WEKA generates a new classifier with optimized hyperparameters, which could be conveniently applied in ordinary WEKA way to the test datasets. The Auto-WEKA deals with hyper-dimensional surface, which has many local minima; therefore, more classifiers could be created with different or same parameters depending on the number and setting of the Auto-WEKA runs. To our knowledge, this is the first application of Auto-WEKA technique to the material science problems.

From Table 2, it can be seen that RF, MP, and Auto-WEKA can easily achieve the 96.6–100% accuracy with 10-fold cross-validation on our training dataset. Note that diagonal and off-diagonal elements in confusion matrix (CM) correspond to the number of correct and incorrect classifications, respectively (see Equation (1)). All other errors and statistical values can be easily calculated from such matrixes:

(1). $$CM=\begin{array}{ccccc} a & b & c & d & \text{\textbackslash{}iddots}\\ aa & ba & ca & da & a\\ ab & bb & cb & db & b\\ ac & bc & cc & dc & c\\ ad & bd & cd & dd & d \end{array}$$

For example, Equations (2)–(5) are the true-positive rate ($TPR_{a}$) or recall, i.e. the proportion of actual positives that are correctly identified; the false-positive rate ($FPR_{b}$), i.e. the proportion of all negatives that still yield positive test outcomes; the positive predictive or precision value ($PPV_{c}$), i.e. the proportions of positive results that are true positive; and the effectiveness measurement ($F-Measure_{d}$), i.e. the weighted average of the $PPV_{c}$ and $TPR_{d}$, for classes a, b, c, and d, respectively:

(2), $$TPR_{a}=TP_{a}/P_{a}=TP_{a}/\left(TP_{a}+FN_{a}\right)=1-FNR_{a}$$

(3), $$FPR_{b}=FP_{b}/N_{b}=FP_{b}/\left(FP_{b}+TN_{b}\right)=1-TNR_{b}$$

(4), $$PPV_{c}=TP_{c}/\left(TP_{c}+FP_{c}\right)$$

(5). $$F-Measure_{d}=2\times PPV_{d}\times TPR_{d}/\left(PPV_{d}+TPR_{d}\right)$$

In Equation (2), the $TP_{a}=aa$, $FN_{a}=ba+ca+da$, $P_{a}=TP_{a}+FN_{a}$, and $FNR_{a}$ are the true-positive (hit), false-negative (miss), condition-positive, and false-negative rate values for class a, respectively, with $FNR_{a}$ to be the proportion of positives which yield negative test outcomes. In Equation 3, the $FP_{b}=ba+bc+bd$, $N_{b}=\sum_{row=a,\neq b}^{d}CM$, $TN_{b}=N_{b}-FP_{b}$, and $TNR_{b}$ are the false-positive (false alarm), number of real-negative cases, true-negative (correct prediction of not belonging to class), and true-negative rate values for class b, respectively, with $TNR_{b}$ to be the proportion of actual negatives that are correctly identified.

Though Auto-WEKA classifiers slightly outperformed the RF and MP ones on the training dataset, but by applying these trained classifiers on the test dataset, the accuracy of correct prediction dropped significantly and differently for all of them with the lowest decrease for RF (see the last column in Table 2). Below, we will demonstrate in more details the overall superiority of RF classifier for our microstructure-labeling problem. Note that increased misclassification on test dataset was mainly due to the instances at 3°C/s cooling rate. Actually, this was the reasonable and good result since these data were obtained from images with insufficient etching to visualize B microstructures. Then, the classifiers mainly assigned these test data to the F/P-type microstructure (b-column in CM), which indicates that our method spotted this problem automatically. The small misclassification between data at 0.3 and 1°C/s cooling rates was also understandable since both samples are F/P-type microstructures and differentiated in F sub-microstructures, i.e. in relative contributions of F_all_, F_gb_, and F_sp_. However, in total, our method can distinguish reasonably well such F/P microstructures at 0.3 and 1°C/s cooling rates. In principle, more precise labeling on the relative contribution of particular F sub-microstructure could be feasible with some attributes from Euclidean distance conversion technique (see, for example, [4]). If to remove 3°C/s data from test dataset, then classification accuracies had improved significantly with all classifiers (see values in parentheses in Table 2). By comparing these values, it can be noted that classifiers with the RF algorithm in their core produced the most accurate predictions.

**Figure 5.**
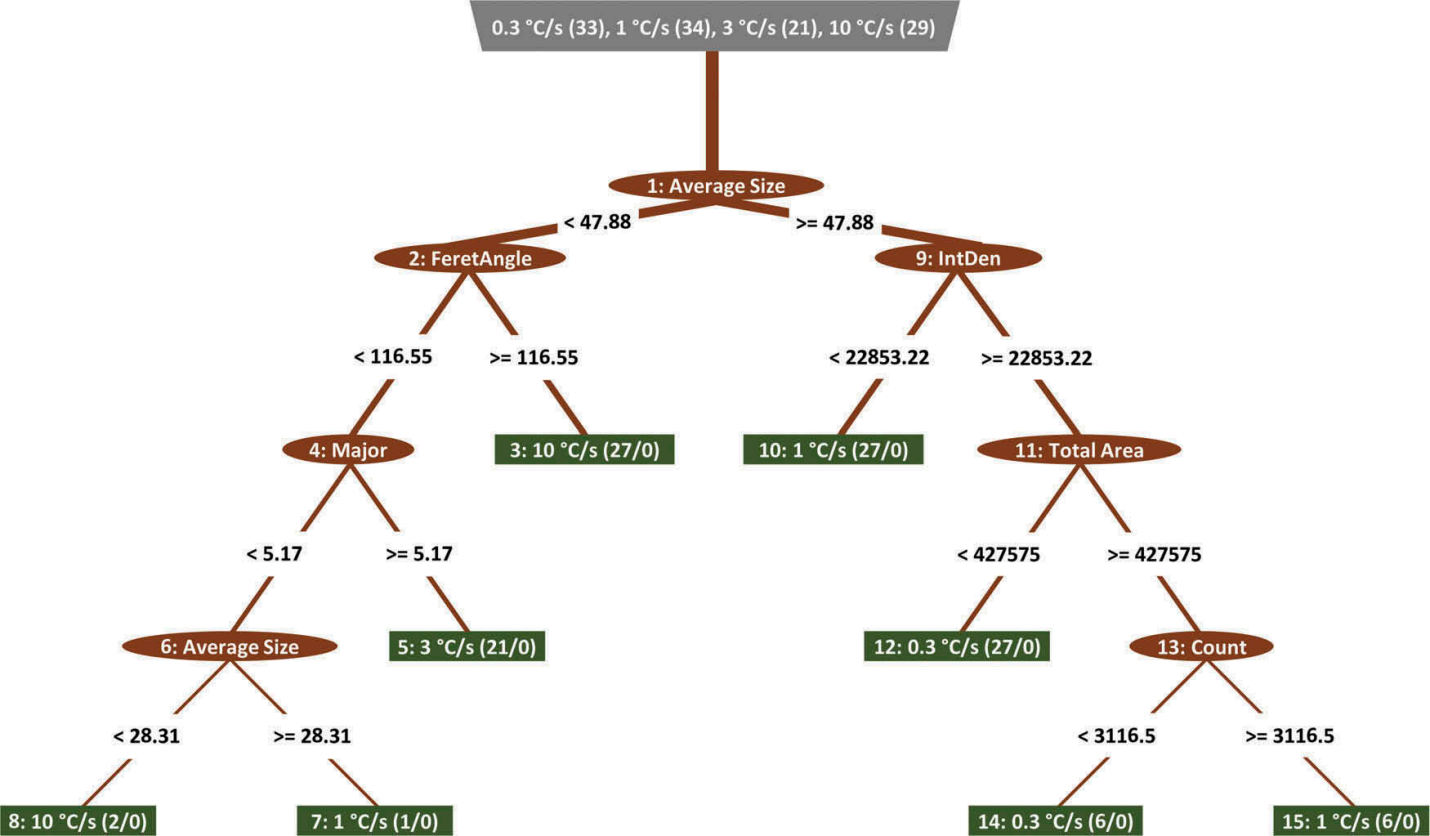
Extracted information from random forest classifier: (a) the attribute importance and (b) the example structure of one tree.

Actually, this was not a surprise. It was reported that by applying 179 classifiers on 121 datasets from the UCI database, the RF classifier versions produced the best results in most of the cases [21]. A similar conclusion was also derived for smaller scale investigation with 65 WEKA classifies on 3 datasets [22]. In our case, the basic RF classifiers even slightly outperformed the Auto-WEKA classifier with RF in its core (98.6 vs. 97.1%). Probably, it was due to only three trees in RF with Auto-WEKA and better internal attribute selection in base RF algorithm compared to greedy stepwise and feature subset selection techniques applied to the training dataset prior to RF classifier creation in Auto-WEKA. Figure 5(a) demonstrates such internal attribute selection in the process of basic RF classifier build-up by plotting of normalized attribute counts from all RF trees. Figure 5(a) also shows that there was little difference between 100 and 1000 trees in the forests, which related to the well-known leveling-off the classification accuracy for forests with more than ~100–200 trees [23,24] and better RF classifier robustness with respect to noise [25]. The attributes, which are irrelevant to our classification task, such as Mode, Mean, FeretX, and FeretY got zero or very low counts in tree nodes due to insignificant statistical effect on information gains in a process of tree growth. Figure 5(a) also demonstrates that RF classifier is not a ‘black box’ since attribute importance could be extracted and each tree has very clear meaning (see Figure 5(b)). The base RF classifier can be also created much faster compared to Auto-WEKA one. Therefore, the automated hyperparameter optimizations still require the careful comparisons. Nevertheless and apart from required PC time and resources, all classifiers listed in Table 2 can produce quite good results with ~86–99% accuracy (see values in parentheses).

**Figure:**
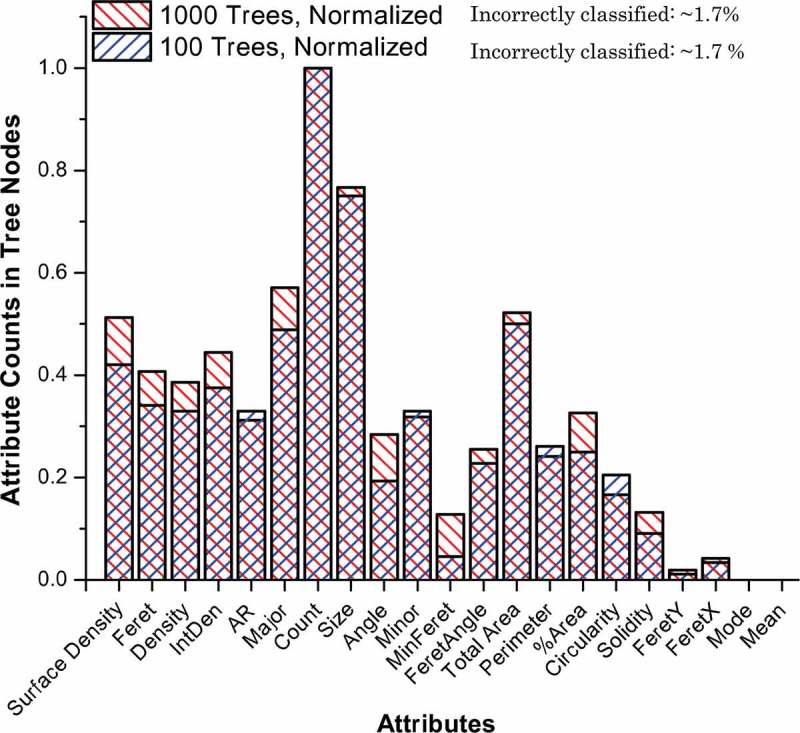

From a practical viewpoint, it is better to do deal with attributes in datasets, which are robust to image scale/area. Otherwise, the images for testing should be collected with the same spatial resolution or additional normalization is necessary. Such robust attributes are the Surface Density, Aspect Ratio, Circularity, and Solidity ones. However, this manual attribute reduction could lead to the decrease in classification accuracy. Table 3 shows the performance of several well-known ML techniques with such reduced training/test datasets. Again, the best classification accuracy was obtained with RF classifier. In this case, the drop in accuracy was only ~1.5% compared to the use of all 21 attributes.

**Table 3.** Performance comparisons between random forest and other popular classifiers. The *a, b, c*, and *d* columns/row labels correspond to the instances of the classification on 0.3, 1, 3, and 10°C/s or (F_gb_+F_all_+F_sp_)/P, (F_gb_+F_all_+F_sp_)/P, (F_all_+F_sp_)/P/P/B, and B/M classes, respectively. The numbers in brackets are calculated by omitting the data at 0.3°C/s. Attributes, which are robust to image scale, were used from Table 1.

Classifier with default settings in WEKA	Ten-fold validation on training dataset	Validation on test dataset
	Confusion matrix, correct classification	Confusion matrix, correct classification
Random forest	$\begin{array}{ccccc} a & b & c & d & \text{.}\text{.}\text{.}\\ 30 & 0 & 0 & 0 & a\\ 0 & 38 & 0 & 0 & b\\ 0 & 3 & 16 & 0 & c\\ 0 & 0 & 0 & 30 & d \end{array}$	$\begin{array}{ccccc} a & b & c & d & \text{.}\text{.}\text{.}\\ 22 & 2 & 0 & 0 & a\\ 0 & 16 & 0 & 0 & b\\ 0 & 16 & 0 & 1 & c\\ 0 & 0 & 0 & 29 & d \end{array}$
	~97.4%	~77.9% (97.1%)
Logistic regression	$\begin{array}{ccccc} a & b & c & d & \text{.}\text{.}\text{.}\\ 29 & 1 & 0 & 0 & a\\ 0 & 38 & 0 & 0 & b\\ 0 & 0 & 19 & 0 & c\\ 0 & 0 & 0 & 30 & d \end{array}$	$\begin{array}{ccccc} a & b & c & d & \text{.}\text{.}\text{.}\\ 24 & 0 & 0 & 0 & a\\ 8 & 8 & 0 & 0 & b\\ 0 & 17 & 0 & 0 & c\\ 0 & 0 & 23 & 6 & d \end{array}$
	~99.1%	~44.2% (55.1%)
Multilayer perceptron (neural networks)	$\begin{array}{ccccc} a & b & c & d & \text{.}\text{.}\text{.}\\ 30 & 0 & 0 & 0 & a\\ 0 & 38 & 0 & 0 & b\\ 0 & 1 & 18 & 0 & c\\ 0 & 0 & 1 & 29 & d \end{array}$	$\begin{array}{ccccc} a & b & c & d & \text{.}\text{.}\text{.}\\ 24 & 0 & 0 & 0 & a\\ 5 & 11 & 0 & 0 & b\\ 0 & 17 & 0 & 0 & c\\ 0 & 0 & 0 & 29 & d \end{array}$
	~98.3%	~74.4% (92.8%)
Naive Bayes	$\begin{array}{ccccc} a & b & c & d & \text{.}\text{.}\text{.}\\ 28 & 2 & 0 & 0 & a\\ 0 & 34 & 4 & 0 & b\\ 0 & 1 & 18 & 0 & c\\ 0 & 0 & 1 & 29 & d \end{array}$	$\begin{array}{ccccc} a & b & c & d & \text{.}\text{.}\text{.}\\ 21 & 3 & 0 & 0 & a\\ 0 & 16 & 0 & 0 & b\\ 0 & 8 & 9 & 0 & c\\ 0 & 0 & 0 & 29 & d \end{array}$
	~93.2%	~87.2% (95.7%)
k-Means	$\begin{array}{ccccc} a & b & c & d & \text{.}\text{.}\text{.}\\ 22 & 8 & 0 & 0 & a\\ 16 & 16 & 6 & 0 & b\\ 1 & 0 & 18 & 0 & c\\ 0 & 0 & 3 & 27 & d \end{array}$	$\begin{array}{ccccc} a & b & c & d & \text{.}\text{.}\text{.}\\ 15 & 9 & 0 & 0 & a\\ 16 & 0 & 0 & 0 & b\\ 3 & 0 & 14 & 0 & c\\ 0 & 0 & 0 & 29 & d \end{array}$
	~70.9%	~67.4% (63.8%)
k-Nearest neighbors	$\begin{array}{ccccc} a & b & c & d & \text{.}\text{.}\text{.}\\ 27 & 3 & 0 & 0 & a\\ 0 & 38 & 0 & 0 & b\\ 0 & 1 & 17 & 1 & c\\ 0 & 0 & 1 & 29 & d \end{array}$	$\begin{array}{ccccc} a & b & c & d & \text{.}\text{.}\text{.}\\ 24 & 0 & 0 & 0 & a\\ 9 & 7 & 0 & 0 & b\\ 1 & 16 & 0 & 0 & c\\ 0 & 0 & 0 & 29 & d \end{array}$
	~94.9%	~69.4% (87.0%)

**Figure 6.**
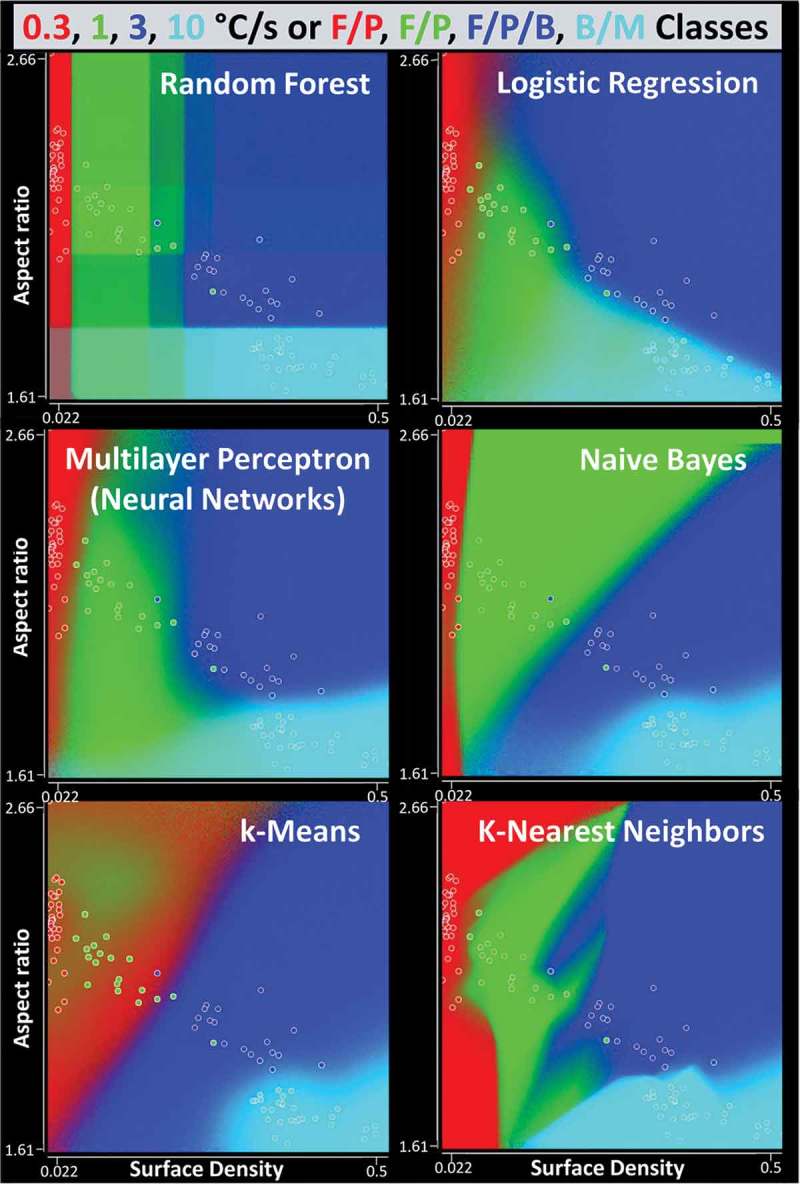
Visualized class decision boundaries for several well-known classifiers (see text and Table 3 for more details).

Figure 6 demonstrates the differences between classifiers in terms of their decision boundaries, which were estimated with smooth interpolations. As indicated by labels, each class maximum probability is assigned with single-color code. Then, RGB pixel color in discretized attribute space is defined by a linear combination of estimated class probabilities (weighted averages based on kernel density estimators), which are calculated by sampling of the points in corresponding attribute space with classification models [26]. Except k-means and k-nearest neighbors classifiers with over-fragmented boundaries, other tested ones have more similarities in this respect. Nevertheless, the RF classifier decision boundaries automatically partitioned the relevant attribute space in the simplest way but in accordance with our intuitively expected distribution of such boundaries for different steel types.

Here, it should be mentioned that Tables 2 and 3 list results with default WEKA settings for all classifiers since they still produced over 90% labeling accuracy on the training dataset, except with k-Means one. Note that these settings are based on known behaviors of these algorithms, i.e. they are not arbitrary for a good start. In principle, the multi-parameter optimizations could be further applied on single classifier from Table 3 with MultiSearch meta-method in WEKA, which could optimize arbitrary number of user-defined parameters and their ranges after attribute selection/filtering with other tools. We did not proceed rigorously with such single classifier tuning due to plausible gains with unjustifiable efforts compared to already achieved high accuracy with default settings for RF algorithm: ~98/99% accuracy on training/test datasets without any external attribute selection/filtering (see Table 2). Nevertheless, these tools could be useful for other training datasets and they are available.

## 4. Conclusions

We have developed the protocol with image processing and ML tools to label the images of steel materials on typical F/P-, F/P/B-, and B/M-type steels. The RF algorithm or its modifications performed overall better compared to other ML tools due to its ensemble, unbiased, and stable nature. Our technique of image attribute extraction and consequent ML application on image datasets could find potential applications in metallurgical research/analytical laboratories.
