# Supplementary figures and images for: Automatic steel labeling on certain microstructural constituents with image processing and machine learning tools
Source: Sci Technol Adv Mater. 2019 Jun 5;20(1):532–42. doi: 10.1080/14686996.2019.1610668 (PMC6567074; doi:10.1080/14686996.2019.1610668)

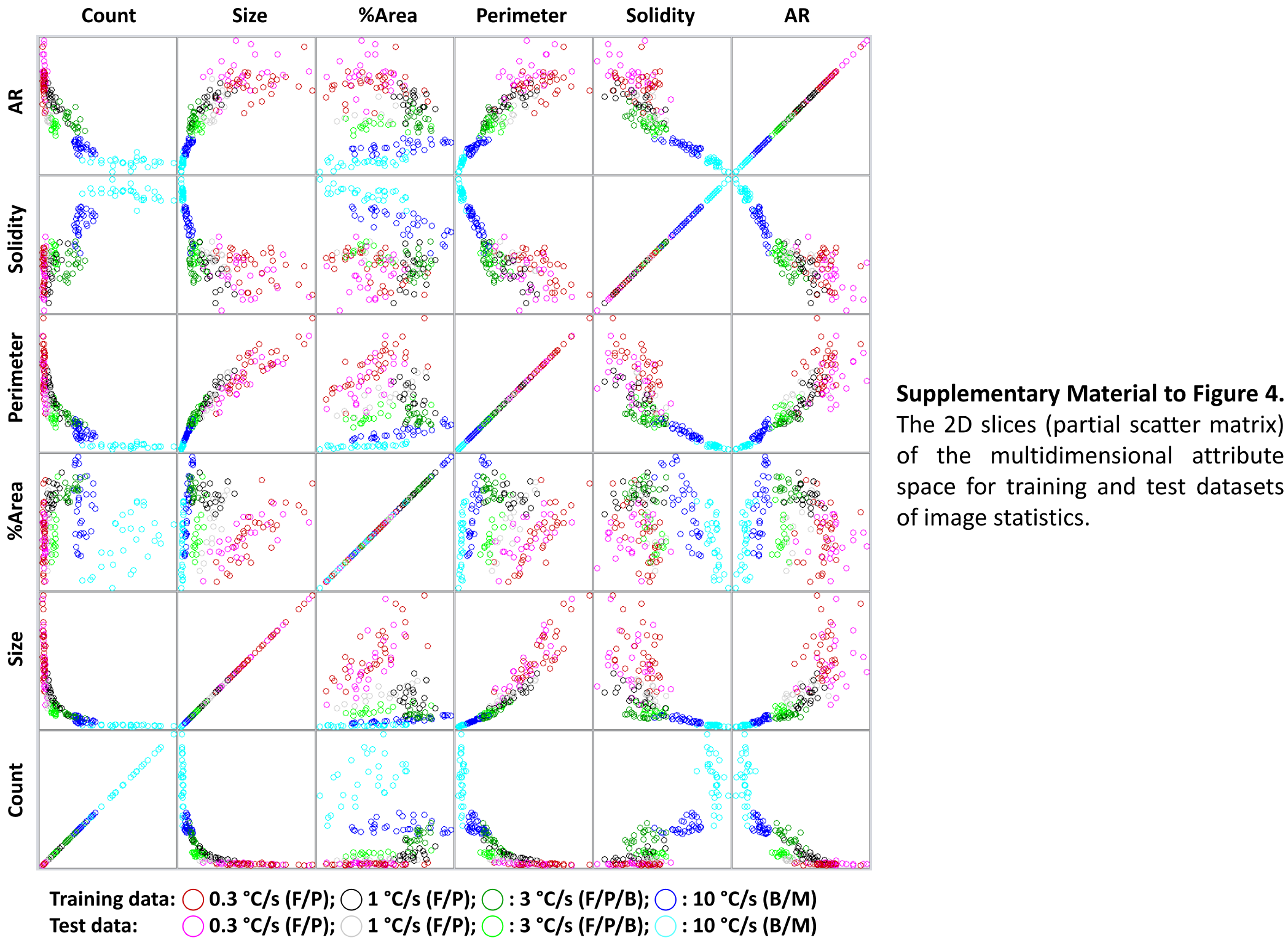

Supplement: Supplemental Material [file TSTA_A_1610668_SM7125.png]
